# Prenatal exposure to trans fatty acids and head growth in fetal life and childhood: triangulating confounder-adjustment and instrumental variable approaches

**DOI:** 10.1007/s10654-022-00910-4

**Published:** 2022-09-15

**Authors:** Runyu Zou, Jeremy A. Labrecque, Sonja A. Swanson, Eric A. P. Steegers, Tonya White, Hanan El Marroun, Henning Tiemeier

**Affiliations:** 1grid.5645.2000000040459992XDepartment of Child and Adolescent Psychiatry, Erasmus MC, University Medical Center Rotterdam, Rotterdam, The Netherlands; 2grid.7692.a0000000090126352Julius Center for Health Sciences and Primary Care, University Medical Center Utrecht, Utrecht University, Utrecht, The Netherlands; 3grid.5477.10000000120346234Institute for Risk Assessment Sciences, Utrecht University, Utrecht, the Netherlands; 4grid.5645.2000000040459992XDepartment of Epidemiology, Erasmus MC, University Medical Center Rotterdam, Rotterdam, The Netherlands; 5grid.21925.3d0000 0004 1936 9000Department of Epidemiology, School of Public Health, University of Pittsburgh, Pittsburgh, PA USA; 6grid.5645.2000000040459992XDepartment of Obstetrics and Gynecology, Erasmus MC, University Medical Center Rotterdam, Rotterdam, The Netherlands; 7grid.5645.2000000040459992XDepartment of Radiology and Nuclear Medicine, Erasmus MC, University Medical Center Rotterdam, Rotterdam, The Netherlands; 8grid.416868.50000 0004 0464 0574Section of Social and Cognitive Developmental Neuroscience, National Institutes of Mental Health, Bethesda, MD USA; 9grid.6906.90000000092621349Department of Psychology, Education and Child Studies, Erasmus School of Social and Behavioral Sciences, Erasmus University Rotterdam, Rotterdam, The Netherlands; 10grid.38142.3c000000041936754XDepartment of Social and Behavioral Sciences, T.H. Chan School of Public Health, Harvard University, Boston, MA 02115 USA

**Keywords:** Trans fatty acids, Pregnancy, Head, Regression analysis, Instrumental variable

## Abstract

**Supplementary Information:**

The online version contains supplementary material available at 10.1007/s10654-022-00910-4.

## Introduction

Trans fatty acids (TFAs) are unsaturated fatty acids with at least one carbon–carbon double bond in the *trans* configuration. The major dietary TFAs are industrially produced to bring plasticity as well as emulsion stability to shortenings and enhance the palatability of baked goods and sweets [1]. Consumption of TFAs can be harmful for human health. Despite the well-documented relations between TFA intake and cardiometabolic consequences such as coronary heart disease and diabetes [2], little evidence has linked TFAs to neurodevelopmental outcomes.

The fetal period is critical for the development of brain structure and functioning, and the fetal brain is vulnerable to environmental adversities [3]. As potential nutritional adversities, TFAs accumulate in the human placenta during pregnancy, and are transported to the fetus in amounts that depend on maternal dietary intake [4]. Higher umbilical TFA levels were associated with a less optimal neurologic status as assessed by a neurological examination in children aged 18 months [5], suggesting that prenatal exposure to TFAs may impact neurodevelopment.

Head circumference (HC) can serve as a proxy for fetal brain development, because the cranial capacity corresponds to brain growth in early life [6]. Moreover, a slower HC growth in utero and smaller HC at birth are related to worse cognitive performance in childhood [7, 8]. In a Dutch cohort that included women in 2008–2009, the researchers reported no association between maternal or umbilical plasma t18:1 concentration and HC at birth [9]. To the best of our knowledge, the association between prenatal TFAs exposure and head size has not been examined using repeated measures.

Most countries rely on food producers to voluntarily reduce the content of industrial TFAs in food, and only a few European Union countries such as Denmark and Iceland have introduced legislative limits. In the Netherlands, large food producers decided to eliminate TFAs from retail products since early 1990s, and by 1996 most retail margarines contained only trace amounts of TFAs. However, fast foods and baked goods remained as the two major sources of TFAs and an important part of the Dutch diet [10]. Starting in 2003, the Margarine, Fats and Oils Product Board (Dutch abbreviation: MVO) initiated the Responsible Fatty Acid Composition Taskforce to further reduce TFA content in Dutch food. This has led to substantial reduction in TFA composition of various materials and products in the Netherlands from 2003 onwards (see Figure S1) [11], and corresponding reduction in TFA intake in the Dutch population has been documented [12].

We aimed to examine the effect of prenatal exposure to TFAs on head growth in fetal life and childhood. We used two complementary methods for effect estimation: regression analysis adjusting for measured confounders, and instrumental variable (IV) analysis. As a clear decline in maternal gestational plasma TFA concentration over the period of recruitment was evident in the current cohort [13], we proposed the calendar time of maternal TFAs assessment as an IV for the relation between maternal TFA status during pregnancy and child head measures. Because confounding bias is a major concern in observational nutrition studies, using these approaches together allows us to better support any causal inferences by triangulating approaches that rest upon different assumptions about confounding. We hypothesized that exposure to higher TFA levels was associated with suboptimal head growth characterized by a smaller HC and lower HC growth rate in fetal life, and lower global brain volume in childhood.

## Methods

### Setting and participants

This study was embedded in the population-based Generation R cohort. Pregnant women with an expected delivery date between April 2002 and January 2006 in Rotterdam were eligible [14]. The Generation R Study was approved by the Medical Ethics Committee of the Erasmus Medical Center, and written informed consent was obtained from adult participants.

A total of 8633 live singletons were born to women recruited in pregnancy. Among these children, 1710 were excluded due to missing data on maternal plasma TFA concentration during pregnancy. After further exclusion of those without ultrasound data on HC in the second or third trimester, 6900 children constituted the study population. Of these, 2933 children underwent a structural brain magnetic resonance imaging (MRI) session at age 9–11 years [15], and 2354 had usable data after quality control (see Figure S2 for the flow-chart).

### Maternal TFA concentration during pregnancy

As previously described [16], maternal plasma fatty acids concentrations were assessed in mid-gestation (mean 20.6 weeks, SD = 1.1) using gas chromatography. The concentrations of individual fatty acids are expressed as weight percentage (%, wt:wt) of all glycerophospholipid fatty acids detected with a chain length between 14 and 22 carbon atoms. For the current study, total TFA concentration was calculated by summing the concentrations of t16:1, t18:1, and tt18:2 isomers. In our sample, the interquartile range (IQR) of total TFA concentration was 0.27–0.41%, and the t18:1 isomer (IQR 0.16–0.28%) accounted for the majority (on average 63%) of total TFAs. Absolute concentration (i.e., mg/L) of TFA was used in a supplementary analysis.

### Head growth in fetal life and childhood

Fetal HC was measured with ultrasound during each trimester of pregnancy by technicians at the Generation R Study research center [14]. First trimester ultrasounds were used for pregnancy dating. In our study population, fetal HC data were available in 6792 children in the second trimester (mean = 20.6 weeks, SD = 1.2), and 6625 children in the third trimester (mean = 30.4 weeks, SD = 1.1). The intra- and inter-observer correlation coefficients for fetal HC were 0.995 and 0.988, suggesting excellent reliability. In addition, we calculated fetal HC growth rate (in cm/week) from the second to third trimester as the difference between HC divided by the difference in gestational age in 6517 children. HC at birth measured using standardized procedures was available in 3752 children [17].

Brain morphology in childhood was assessed using MRI at age 9–11 years. All images were acquired on a 3-Tesla GE Discovery MR750w MRI System (General Electric, Milwaukee, WI, USA) scanner using an 8-channel head coil. High-resolution T_1_-weighted sequences were obtained using a 3D coronal inversion recovery fast spoiled gradient recalled (IR-FSPGR, BRAVO) sequence with 1-mm isotropic resolution, and images were rated for quality control both during and after the MRI acquisition [15]. Volumetric segmentation and cortical reconstruction were performed with FreeSurfer v.6.0.0 (http://surfer.nmr.mgh.harvard.edu/), and the standard reconstruction stream was applied. The quality of FreeSurfer output was visually inspected, and data with insufficient quality were eliminated. For the current study, total brain volume, and volumes of cortical gray matter and cerebral white matter were used to quantify global brain size.

### Covariates

We included the following maternal and child characteristics as covariates based on prior literature [13, 18]: child sex and (gestational) age at head assessment, maternal ethnicity, age at enrollment, pre-pregnancy body mass index (BMI), psychopathology, marital status, parity, educational level, total energy intake, diet quality, smoking and alcohol use during pregnancy, and family income. Crown-rump length or bi-parietal diameter was used to determine gestational age. Maternal psychopathology was assessed using the Brief Symptom Inventory (scores range from 0 to 4, with higher scores indicating more clinically relevant psychological symptoms) [19]. Maternal diet quality was assessed in early pregnancy using a semi-quantitative 293-item food frequency questionnaire and quantified by an overall score ranging from 0 to 15, with higher score reflecting better adherence to Dutch dietary guidelines [20].

### Additional variables

**Fig. 1 Fig1:**
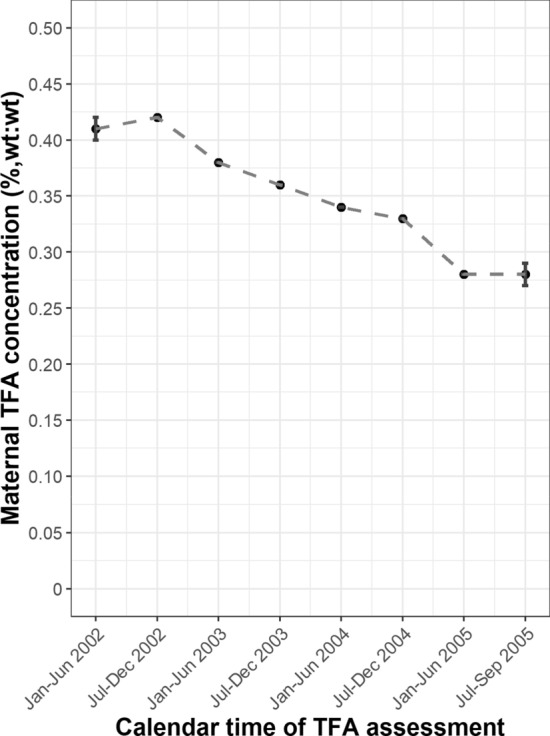
Maternal trans fatty acid concentration during pregnancy per calendar time of assessment. Maternal TFA concentration (%, wt:wt, mean ± se) per calendar time of TFA assessment. Maternal TFA concentration was assessed in the plasma at 20.6 (1.1) weeks of gestation. The Dutch initiative to further reduce TFA content in food took effect in 2003. Abbreviation: TFA, Trans fatty acid

Information on calendar time of maternal TFA assessment during pregnancy was used as an IV for the IV analysis. The recruitment of pregnant women approximately concurred with the initiative on TFAs reduction (see Fig. 1 and Figure S1).

Given the evidence suggesting a relation between prenatal HC and childhood cognitive development [7, 8], child nonverbal IQ assessed at age 5–9 (mean = 6.2) years was used in a secondary analysis [21]. We examined the association of HC in utero with cognitive abilities in childhood in our sample to highlight the clinical implications of our findings.

### Statistical analysis

For descriptive purposes, continuous variables were presented as mean (SD) and categorical variables as number (%). In a non-response analysis, we describe the characteristics of children with and without MRI data at 9–11 years, using analysis of variance (ANOVA) or Wilcoxon test for continuous variables, and chi-square tests for categorical variables.

We investigated the association of maternal TFA concentration with HC in second and third trimesters, fetal HC growth rate, and global brain volume (i.e., total brain volume, cortical gray matter volume, and cerebral white matter volume) at age 9–11 years using linear regression. We also examined maternal TFA concentration in relation to HC at birth in a supplementary analysis. These regression analyses were performed using multi-stage models with different levels of confounder adjustment, to explore how adjustment for measured confounding affects estimates. In the initial model, only child sex and (gestational) age at outcome assessment were adjusted for. Using a change-in-estimate criterion of 5% [22], maternal ethnicity, age at enrollment, educational level, diet quality, smoking during pregnancy, and family income were selected from the covariate pool as confounders and were additionally adjusted for in a second model. In a third model, we additionally corrected for concentrations of maternal essential fatty acids (EFAs, i.e., α-linoleic acid and linoleic acid) and long-chain polyunsaturated fatty acids (LC-PUFAs), because they can be either confounders (i.e., reductions of TFA may increase PUFAs in body fat composition) or intermediates (i.e., TFA may interfere with EFAs and reduce the synthesis of LC-PUFAs) [23–25]. Further, models with MRI measures were weighted by inverse probability weights to account for attrition (see supplementary methods for details). We examined non-linear relations by comparing the linear model and the model including splines using likelihood ratio test.

To explore implications on long-term neurocognitive development, we investigated prenatal exposure to TFA, and HC and HC growth rate in utero in relation to nonverbal IQ at age 6 years in a secondary analysis.

Next, for head outcomes that were substantially associated with maternal TFA concentration using confounder-adjustment approaches, we performed an IV analysis using two-stage least squares (TSLS) estimation [26]. Calendar time of maternal TFA assessment was proposed as the IV given the initiative to reduce food TFA content in the Netherlands since 2003. In addition, we assessed the basic assumptions of IV analysis (see supplementary methods for details) to evaluate whether calendar time of maternal TFA assessment could be utilized as an IV. For the ‘relevance’ assumption, we examined the association between calendar time of maternal TFA assessment and maternal TFA concentration, both with and without adjusting for covariates. In addition, we performed an F-test on the instrument in the first stage of the TSLS regression. Since the ‘exclusion restriction’ and ‘exchangeability’ assumptions cannot be verified, we explored our rich observed data in ways that might falsify these assumptions. These analyses were performed to minimize the risk that a covariate with a similar time trend as maternal TFA concentration underlies the relation between calendar time of maternal TFA assessment and child head measures. First, we related calendar time of maternal TFA assessment to each covariate using separate linear or logistic regression model, and covariates that did not vary by calendar time of maternal TFA assessment were excluded. Second, the mean ± standard error (se) of each remaining covariate over calendar time of maternal TFA assessment was plotted to inspect time trends. In the primary IV analysis, we adjusted for no covariates, and the sandwich estimator was used because it is robust to heteroscedastic errors. In the secondary analysis, we conducted two TSLS regressions to rule out bias caused by any measured nuisance variables: first, we adjusted for covariates with a potential monotonic time trend; second, we adjusted for all covariates regardless their variation over calendar time. Since maternal TFA concentration was the dependent variable in the first-stage model of the IV analysis, we log-transformed the raw values to obtain a normal distribution. Finally, because we are interested in seeing how the IV analysis results complement the confounding-adjustment regression results, we interpret our IV analyses as primarily testing a causal null hypothesis [27].

We performed two sensitivity analyses to test the robustness of our findings. First, we re-ran the analysis in participants of Dutch national origin only. Second, we examined the trans 18:1 fatty acid, the primary TFA isomer, as exposure of interest.

Missing data on covariates were accounted for by multiple imputation. We generated 20 imputed datasets with 20 iterations, and report pooled results. Statistical significance was set as α < 0.05 (two-sided), and a false discovery rate (FDR) correction was performed in primary analyses [28]. All statistical analyses were performed using R version 3.6.2 (R Foundation for Statistical Computing, Vienna, Austria), including the ‘ivpack’ package for IV analysis.

## Results

### Descriptive

Table 1 shows the characteristics of the participating mother–child dyads. Enrolled at an average age of 29.8 years, nearly half of the mothers were of Dutch national origin, and the majority (87.6%) had completed secondary education or above. Compared to children with MRI data, those without MRI data were born to younger and less highly educated women, more likely had parents with less income, and were exposed to higher maternal TFA concentration during pregnancy (see Table S1).

**Table 1 Tab1:** Descriptive information (n = 6900)

Characterisitcs	Statistics
*Maternal*	
Age at enrollment, years, mean (SD)	29.8 (5.2)
*Ethnicity, n (%)*	
Dutch	3371 (48.9)
Non-Dutch western	569 (8.2)
Non-Dutch non-western	2960 (42.9)
Marital status (married/with partner), n (%)	5858 (84.9)
Pre-pregnancy BMI, mean (SD)	23.8 (4.4)
Psychopathology, score, mean (SD)^a^	0.3 (0.4)
Parity (nullipara), n (%)	3853 (55.8)
*Educational level, n (%)*	
Primary or below	854 (12.4)
Secondary	3249 (47.1)
Higher	2797 (40.5)
*Smoking during pregnancy, n (%)*	
Never smoked	4980 (72.2)
Smoked until pregnancy was known	622 (9.0)
Continued smoking	1298 (18.8)
*Alcohol use during pregnancy, n (%)*	
Never drank	3281 (47.6)
Drank until pregnancy was known	918 (13.3)
Continued drinking occasionally	2207 (32.0)
Continued drinking frequently	494 (7.2)
Total energy intake, kcal, mean (SD)	2018.0 (568.8)
Diet quality, score, mean (SD)^b^	7.5 (1.7)
*Family income, €/month, n (%)*	
< 1200	1750 (25.4)
1200–2000	1358 (19.7)
> 2000	3792 (55.0)
*Child*	
Sex (male), n (%)	3487 (50.5)
Age at neuroimaging, years, mean (SD)^c^	10.1 (0.6)

BMI, body mass index

Data from the first imputed dataset are reported. Percentages have been rounded and may not total 100

^a^Assessed using the Brief Symptom Inventory. Scores range from 0 to 4, with higher scores indicating more clinically relevant psychological symptoms

^b^Assessed using a food frequency questionnaire. The overall score ranges from 0 to 15, with higher scores reflecting better adherence to Dutch dietary guidelines

^c^Data available in 2354 subjects

### Prenatal exposure to TFA and HC in fetal life

The mean difference in HC in the second trimester per 1%, wt:wt increase in maternal TFAs was 0.07 (95% CI − 0.05, 0.19) cm, with similar results for different levels of confounding adjustment (Table 2). However, there was a strong difference in the third trimester, namely a 1%, wt:wt higher TFA concentration of the mother corresponded to 0.33 (95% CI 0.51, 0.15) cm lower HC of the fetus. Higher maternal TFA levels during pregnancy were also related to lower HC growth rate, namely per 1%, wt:wt increase in TFA concentration of the mother corresponded to 0.04 (95% CI: 0.06, 0.02) cm/week slower HC growth of the fetus. Both associations survived FDR correction. Consistent findings were obtained using log-transformed or absolute concentration of maternal TFA (see Table S2-S3). Despite a positive trend, the association of prenatal exposure to TFA with HC at birth was not definite after full adjustment for covariates (B = 0.4, 95% CI −0.04, 0.8, cm). We found no evidence for non-linear relations (see Table S4).

**Table 2 Tab2:** Maternal trans fatty acid concentration during pregnancy in relation to fetal head circumference and head circumference growth

Maternal TFA concentration	Fetal HC at single assessments^a^						Fetal HC growth rate across assessments^b^ (n = 6517)		
	Second trimester (n = 6792)			Third trimester (n = 6625)					
	B	95% CI	*p*-value	B	95% CI	*p*-value	B	95% CI	*p*-value
Model 1	0.13	0.01, 0.24	0.03	− 0.26	− 0.43, − 0.08	0.004	− 0.04	− 0.05, − 0.02	< 0.001
Model 2	0.12	0.004, 0.24	0.04	− 0.30	− 0.47, − 0.12	< 0.001	− 0.04	− 0.06, − 0.02	< 0.001
Model 3	0.07	− 0.05, 0.19	0.24	− 0.33	− 0.51, − 0.15	< 0.001^*^	− 0.04	− 0.06, − 0.02	< 0.001^*^

*TFA* Trans fatty acid, *HC* Head circumference

^a^B’s represent difference in fetal HC (cm) per 1%, wt:wt increase in maternal TFA concentration. Model 1 was adjusted for child sex and gestational age at HC assessment; Model 2 was additionally adjusted for maternal ethnicity, age at enrollment, educational level, diet quality, smoking during pregnancy, and family income; Model 3 was additionally adjusted for maternal concentrations of essential fatty acids and long-chain polyunsaturated fatty acids

^b^B’s represent difference in fetal HC growth rate (cm/week) per 1%, wt:wt increase in maternal TFA concentration. Model 1 was adjusted for child sex. Model 2 was additionally adjusted for maternal ethnicity, age at enrollment, educational level, diet quality, smoking during pregnancy, and family income. Model 3 was additionally adjusted for maternal concentrations of essential fatty acids and long-chain polyunsaturated fatty acids

*These *p*-values survived a false discovery rate (FDR) correction for multiple comparison

### Prenatal exposure to TFA and MRI measures at age ten years

In children with MRI assessments at age 10 years, after full adjustment for covariates, we observed an increase in child total brain volume per 1%, wt:wt higher maternal TFA concentration (mean = 7.8 cm^3^, 95% CI −24.7, 40.3, not weighted by inverse probability; and 21.5 cm^3^, 95% CI −13.2, 56.3, weighted by inverse probability). However, the wide confidence intervals suggest these are inconclusive associations. Similar findings were obtained when relating maternal TFA concentration during pregnancy to cortical gray or cerebral white matter volume of the child (see Table 3).

**Table 3 Tab3:** Maternal trans fatty acids concentration during pregnancy in relation to child brain volumes at age 9–11 years

Maternal TFA concentration	Total brain volume			Cortical gray matter volume			Cerebral white matter volume		
	B	95% CI	*p*-value	B	95% CI	*p*-value	B	95% CI	*p*-value
*Without IPW*									
Model 1	32.1	− 0.8, 65.0	0.06	19.1	2.6, 35.6	0.02	9.6	− 5.3, 24.6	0.21
Model 2	7.9	− 24.4, 40.1	0.63	6.7	− 9.4, 22.8	0.41	2.0	− 13.0, 16.9	0.80
Model 3	7.8	− 24.7, 40.3	0.64	6.5	− 9.7, 22.8	0.43	2.4	− 12.7, 17.4	0.76
*With IPW*									
Model 1	41.9	8.5, 75.4	0.01	23.4	6.6, 40.2	0.006	14.6	− 0.9, 30.1	0.06
Model 2	21.0	− 13.3, 55.3	0.23	13.1	− 4.5, 30.6	0.14	8.0	− 7.8, 23.9	0.32
Model 3	21.5	− 13.2, 56.3	0.22	13.5	− 4.4, 31.3	0.14	8.3	− 7.7, 24.4	0.31

*TFA* Trans fatty acid, *IPW* Inverse probability weighting (used to account for attrtion)

B’s represent volumetric difference (cm^3^) per 1%, wt:wt increase in maternal TFA concentration. Model 1 was adjusted for child sex and age at neuroimaging. Model 2 was additionally adjusted for maternal ethnicity, age at enrollment, educational level, diet quality, smoking during pregnancy, and family income. Model 3 was additionally adjusted for maternal concentrations of essential fatty acids and long-chain polyunsaturated fatty acids. The sample size for all analyses was 2354

### IV analysis

The calendar time of maternal TFA assessment ranged from January 2002 to September 2005. Figure 1 visualizes the change of maternal TFA concentration (mean ± se) over time. There was an inverse, overall monotonic relation between calendar time of TFA assessment and TFA concentration, except in the first few months (see Figure S3 for logarithmically transformed values).

The calendar time of maternal TFA assessment was inversely associated with maternal TFA concentration (mean reduction in %, wt:wt per month, without adjustment for covariates: −0.0108, 95% CI −0.0115, −0.0101; after adjustment for covariates: − 0.0104, 95% CI −0.0111, −0.0097), and the heteroskedasticity-robust F-statistic for calendar time of TFA assessment in the covariate-adjusted model was 971.8 (*p* < 0.001), supporting the ‘relevance’ assumption. Next, we examined potential violation of the ‘exclusion restriction’ and ‘exchangeability’ assumptions; child sex and gestational age at ultrasound, maternal ethnicity, and family income were not predicted by calendar time of TFA assessment. Figure S4 shows variations of maternal age at enrollment, educational level, diet quality, and smoking during pregnancy over calendar time of maternal TFA assessment, with the most plausible monotonic time trend observed in diet quality.

Table 4 shows the results of IV analysis. Higher maternal TFA concentration during pregnancy was associated with smaller fetal HC in the third trimester and lower HC growth rate from the second to the third trimester in the unadjusted model (Model 1). Consistent findings were obtained in analyses adjusting for covariates with time variations only (Model 2) or all covariates (Model 3), suggesting robust findings.

**Table 4 Tab4:** Instrumental variable analysis of maternal trans fatty acids concentration during pregnancy in relation to fetal HC in the third trimester and fetal HC growth rate across assessments

Model	Fetal HC in the third trimester^a^			Fetal HC growth rate across assessments^b^		
	B	95% CI	*p*-value	B	95% CI	*p*-value
1	− 0.77	− 1.0, − 0.51	< 0.001	− 0.11	− 0.13, − 0.09	< 0.001
2	− 0.66	− 0.91, − 0.40	< 0.001	− 0.10	− 0.12, − 0.08	< 0.001
3	− 1.0	− 1.2, − 0.79	< 0.001	− 0.11	− 0.14, − 0.09	< 0.001

*HC* Head circumference

Instrumental variable analysis on maternal trans fatty acids concentration during pregnancy in relation to fetal HC in the third trimester (n = 6383) and HC growth across assessments in the second and third trimesters (n = 6280) was performed using two-stage least squares estimation. Calendar time of maternal trans fatty acids assessment was used as the instrumental variable. The raw values of maternal trans fatty acids concentration were log-transformed to obtain a normal distribution

^a^B’s represent difference in fetal HC (cm) per one unit increase in natural logarithm transformed maternal trans fatty acids concentration. Model 1 was adjusted for no covariates. Model 2 was adjusted for maternal age at enrollment, educational level, diet quality, and smoking during pregnancy. Model 3 was adjusted for all covariates, including gestational age at ultrasound, child sex, maternal ethnicity, age at enrollment, educational level, diet quality, smoking during pregnancy, family income, and maternal concentrations of essential fatty acids and long-chain polyunsaturated fatty acids

^b^B’s represent difference HC growth rate (cm/week) per one unit increase in natural logarithm transformed maternal trans fatty acids concentration. Model 1 was adjusted for no covariates. Model 2 was adjusted for maternal age at enrollment, educational level, diet quality, and smoking during pregnancy. Model 3 was adjusted for all covariates, including child sex, maternal ethnicity, age at enrollment, educational level, diet quality, smoking during pregnancy, family income, and maternal concentrations of essential fatty acids and long-chain polyunsaturated fatty acids

Analyses in participants of Dutch national origin or examining the trans 18:1 fatty acid isomer yielded results similar to the primary analyses (see Table S5-S8, Figure S5-S6).

### TFA concentration during pregnancy and HC in fetal life in relation to nonverbal IQ in childhood

As shown in Table S9, we observed no association between prenatal TFA exposure and nonverbal IQ at age 6 years after full adjustment for covariates. However, both HC in the third trimester and HC growth rate between the second and third trimesters were positively related to nonverbal IQ at year 6 years.

## Discussion

In this population-based study, a higher gestational TFA level was associated with a smaller fetal HC in the third trimester and lower fetal HC growth rate from the second to the third trimester. Analyses utilizing the fact that average TFA concentration in maternal plasma decreased over the years of enrollment showed consistent results. We found no clear association between prenatal exposure to TFAs and fetal HC in the second trimester or global brain volume at age 10 years.

A few studies related maternal TFAs during pregnancy to birth outcomes such as birth length and birth weight [13, 29], but the association between exposure to TFAs during pregnancy and head growth has rarely been examined. Dirix et al. [9, 30] found neither maternal plasma t18:1 content during pregnancy or at delivery, nor umbilical cord plasma t18:1 concentration was related to neonatal HC. The null association between maternal TFA concentration during pregnancy and child HC at birth was replicated in the current study. However, it is important to note that postnatal HC measurements also include skin and subcutaneous tissue. Further, the accuracy of postnatal HC is lower and influenced by the measuring tape [31]. To the best of our knowledge, there have been no studies associating prenatal TFAs exposure with sonographic HC in utero or child brain morphology.

Our study suggests that the smaller HC in the third trimester of the fetus exposed to higher maternal TFA levels during mid-pregnancy could be attributed to the slower HC growth from the second to the third trimester. This association was not accounted for by maternal EFAs or LC-PUFAs concentrations. Several other explanations are possible. First, randomized clinical trials (RCTs) and observational studies indicated that TFAs are associated with systematic inflammation, characterized by elevated C-reactive protein (CRP) and pro-inflammatory cytokines such as tumor necrosis factor-α (TNF-α) and interleukin 6 (IL-6) [32]. Smaller brains in mice offspring exposed to maternal immune activation during pregnancy have been reported [33]. Second, considerable epigenomic changes take place in the human brain during fetal brain development. Robinson et al. [34] found maternal TFAs at preconception and in pregnancy were related to hypomethylation of about 50% of CpG sites in the newborn. Similarly, high TFA doses were related to global hypomethylation in a rodent model [35].

Despite the HC differences in the third trimester, associations between prenatal exposure to TFA levels and global brain size at age 10 years were inconclusive. An absence of differences in brain size may be explained by catch-up head growth after birth, which has been reported in preterm birth and/or small-for-gestational-age term infants [36, 37]. Given the inherent brain plasticity, favorable environmental inducers such as breastfeeding and healthy diet in childhood may gradually revert the differences in brain size in the third trimester due to TFAs exposure [38]. Moreover, the sample size for the MRI analyses was roughly 1/3 of that for the HC analyses, limiting the ability to detect subtle differences.

Causal inference is challenging in most nutritional epidemiological studies using an observational design because of unmeasured or residual confounding often related to other lifestyle choices and socio-economic factors [39]. RCTs would provide rigorous evidence for causal effect estimation but are frequently not feasible or ethical to study the impact of TFAs. In observational studies, coupling confounder-adjustment analyses with IV analysis can help elucidate the effects of diet or nutrients on health because they rely on different assumptions than the no-unmeasured-confounding assumption of common observational study analyses [40]. In the current study, IV and regression analyses allowed for estimating the effect of maternal TFAs on child head measures under distinct assumptions. The agreement between the results of the regression and IV analyses strengthens the possibility that the association between TFA exposure and fetal head growth is causal [41]. This triangulation integrated results from different approaches with different possible sources of bias. Furthermore, we performed analyses adjusting for covariates in the TSLS regression to support our findings. In these analyses, even covariates with a less clear monotonic time trend were included to limit subjective confounder selection. For example, maternal smoking during pregnancy was adjusted for given the strong evidence suggesting its impact on fetal head growth [42], despite an unclear monotonic pattern of variation over calendar time.

Our findings have important public health implications, because WHO aims at global elimination of industrially produced TFAs by 2023, yet industrial TFA content in food remains high, in particular, in South Asia (e.g., India), and Eastern and South-eastern European countries [43, 44]. Statutory measures to reduce TFA intake are uncommon, even in the USA or the European Union. Moreover, even in countries that booked successes in reducing TFAs, high levels of TFA can still be found in certain foods (e.g., biscuits) [45]. Therefore, pregnant women living in countries/regions with high TFA content in food, or those with a preference for food containing high TFA levels have substantially elevated risk of exceeding TFA intake. The smaller fetal HC related to higher TFA exposure may have clinical implications. Previous studies associated lower HC in utero with suboptimal neurodevelopment such as more autistic traits and sleep problems in childhood [46, 47]. The current analyses show that children with a smaller HC in utero also had worse cognitive abilities at age 6 years. Therefore, reduction on maternal TFA intake during pregnancy likely favors offspring neurodevelopment.

Several limitations should be acknowledged. First, we did not assess maternal TFA status in early pregnancy when the basis of embryonal neurodevelopment is substantially formed. However, because the diet patterns of women is reasonably stable across pregnancy [48], maternal TFA levels in mid-gestation were likely indicative of those in the earlier stages. Second, we examined the primary TFA isomer, but were not able to examine other specific isomers, such as t9-C18:1t and t10-C18:1t. However, there is no evidence for a specific mechanism of action [49]. Third, concerns about the violation of some IV assumptions in nutritional research have been raised because nutrition is correlated with numerous other lifestyle and environmental factors [50]. In the current study, we accounted for various variables in the IV analysis, such as EFAs, LC-PUFAs, and diet quality that might index overall nutritional status. However, residual confounding by other nuisance variables, especially those with a similar time trend (e.g., specific nutrients used to replace the TFAs) that may attenuate the observed relations, could not be ruled out.

To conclude, exposure to higher TFA levels during pregnancy is related to lower HC in the third trimester and slower HC growth from the second to the third trimester. These findings further justify advocating TFAs reduction across the globe in the pursuit of optimal child development.

## References

## Supplementary Information

Below is the link to the electronic supplementary material.Supplementary file1 (DOCX 1055 KB)
