# Supplementary material for: Prenatal exposure to trans fatty acids and head growth in fetal life and childhood: triangulating confounder-adjustment and instrumental variable approaches
Source: Eur J Epidemiol. 2022 Sep 15;37(11):1171–80. doi: 10.1007/s10654-022-00910-4 (PMC9755085; doi:10.1007/s10654-022-00910-4)
Supplement: Supplementary file 1 — Supplementary file1 (DOCX 1055 KB) [file 10654_2022_910_MOESM1_ESM.docx]

**Figure S1. Trans fatty acid content (% on fat basis) changes in the Netherlands**


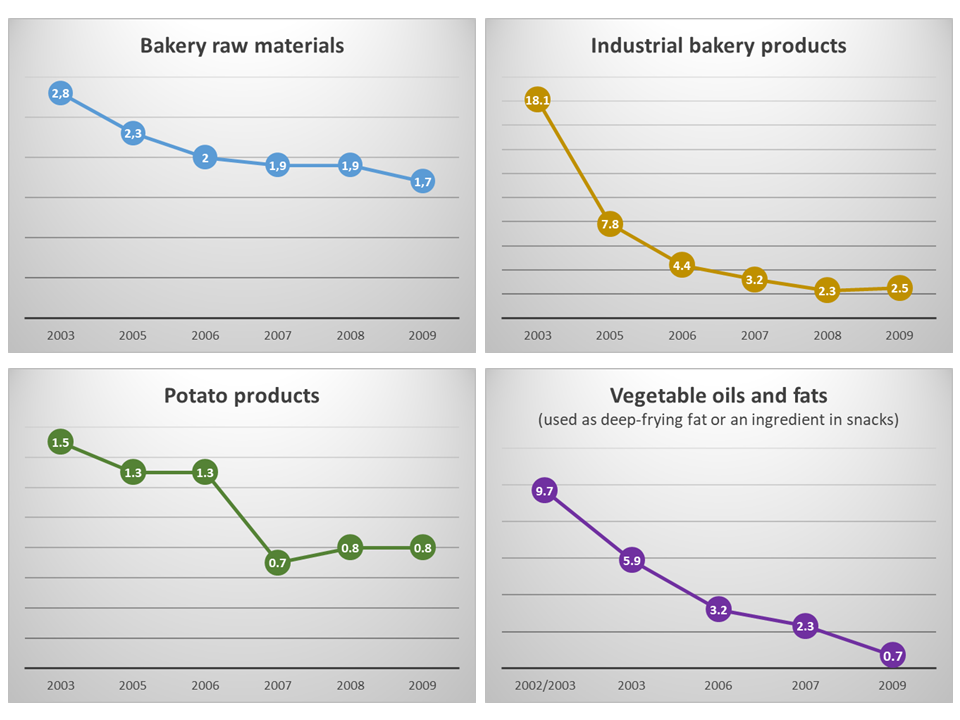


Data source: Website Task Force Verantwoorde Vetzuursamenstelling [website]. Rijsvijk: Task Force Verantwoorde Vetzuursamenstelling; 2015. (<https://mvo.nl/themas/voeding-gezondheid/taskforce-verantwoorde-vetzuursamenstelling>, accessed 12 November, 2021).

**Figure S2. Flow-chart of study population selection**

8633 live singletons born to women recruited since pregnancy

6900 with fetal ultrasound HC from mid-gestation onward

- 6792 in the second trimester
- 6625 in the third trimester

6923 with information on maternal plasma TFAs in mid-gestation

1710 children without information on maternal plasma TFAs assessed in mid-gestation were excluded

23 children without any data on fetal ultrasound HC from mid-gestation onward were excluded

2354 children with usable MRI data at 9-11 years

3967 children were excluded due to loss to follow-up or absence of brain MRI assessment at 9-11 years

2933 children underwent brain MRI with consent at 9-11 years

579 children were excluded due to heterogeneous scanner parameters, major incidental findings, artifacts, and/or insufficient image quality

**Supplementary Methods**

Calculation of inverse probability weights:

Inverse probability weights were calculated using binary logistic regression with respondent (1) or non-respondent (0) as the dependent variable, and maternal trans fatty acids concentration, child sex, maternal ethnicity, age at enrollment, marriage status, pre-pregnancy BMI, psychopathology, parity, educational level, energy intake, diet quality, smoking and alcohol use during pregnancy, and family income as predictors. In addition, weights stabilization was performed by replacing the numerator (i.e., 1 in the unstabilized weights) with the probability of being included in the analysis of neuroimaging data at age ten years (i.e., 2354/6900), to avoid extreme weight values that tend to increase the variance of the effect estimate.

Three key assumptions for instrumental variable (IV) analysis:

1) the instrument Z is associated with the exposure X (‘relevance’)

2) Z affects the outcome Y only through X (‘exclusion restriction’)

3) Z does not share common causes with Y (‘exchangeability’).

A valid IV analysis can provide estimate free of bias due to unmeasured confounding factors U (see below the illustrative causal diagram).


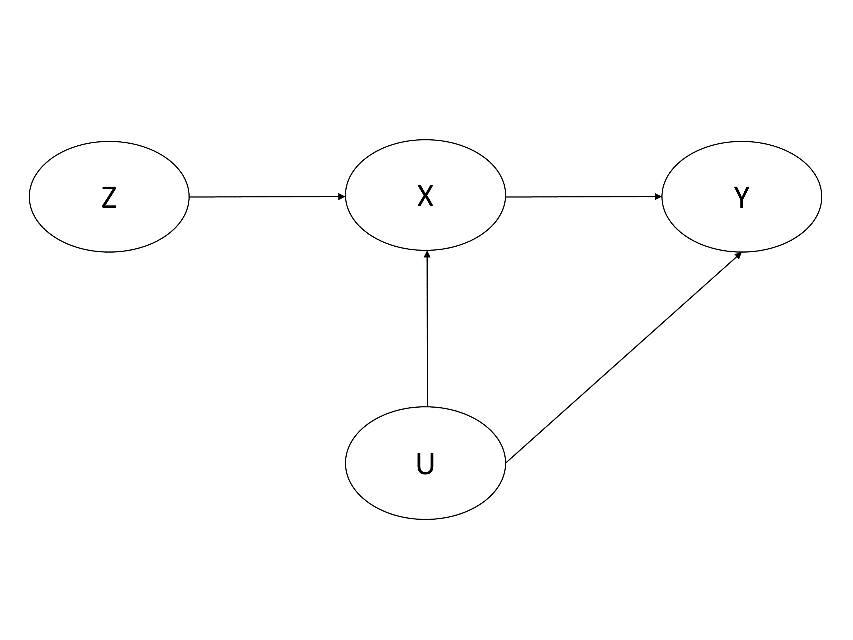


X-exposure variable (i.e., maternal trans fatty acid concentration in this study);

Y-outcome variable (i.e., fetal head circumference);

Z-instrumental variable (i.e., calendar time of maternal TFA assessment);

U-measured or unmeasured confounding variables.

**References**

Chesnaye NC, Stel VS, Tripepi G, Dekker FW, Fu EL, Zoccali C, Jager KJ. An introduction to inverse probability of treatment weighting in observational research. Clin Kidney J. 2021;15(1):14-20.

Greenland S. An introduction to instrumental variables for epidemiologists. Int J Epidemiol. 2000;29(4):722-9.

Labrecque J, Swanson SA. Understanding the Assumptions Underlying Instrumental Variable Analyses: a Brief Review of Falsification Strategies and Related Tools. Curr Epidemiol Rep. 2018;5(3):214-220.

**Table S1. Non-response analysis comparing children with and without MRI data at age 9-11 years**

| **Characteristics** | **Children with MRI**  **(n=2354)** | **Children without MRI**  **(n=4546)** | **p-value** |
| --- | --- | --- | --- |
| **Maternal** |  |  |  |
| Age at enrollment, years, mean (SD) | 30.9 (4.7) | 29.2 (5.4) | <0.001 |
| Ethnicity, n (%) |  |  |  |
| Dutch | 1381 (58.7) | 1990 (43.8) | <0.001 |
| Non-Dutch western | 183 (7.8) | 386 (8.5) |  |
| Non-Dutch non-western | 790 (33.6) | 2170 (47.7) |  |
| Marital status (married/with partner), n (%) | 2085 (88.6) | 3773 (83.0) | <0.001 |
| Pre-pregnancy BMI, mean (SD) | 23.5 (4.1) | 23.9 (4.6) | 0.001 |
| Psychopathology, score, mean (SD)^a^ | 0.3 (0.3) | 0.3 (0.4) | <0.001 |
| Parity (nullipara), n (%) | 1416 (60.2) | 2437 (53.6) | <0.001 |
| Educational level, n (%) |  |  |  |
| Primary or below | 179 (7.6) | 675 (14.8) | <0.001 |
| Secondary | 975 (41.4) | 2274 (50.0) |  |
| Higher | 1200 (51.0) | 1597 (35.1) |  |
| Smoking during pregnancy, n (%) |  |  |  |
| Never smoked | 1792 (76.1) | 3188 (70.1) | <0.001 |
| Smoked until pregnancy was known | 222 (9.4) | 400 (8.8) |  |
| Continued smoking | 340 (14.4) | 958 (21.1) |  |
| Alcohol use during pregnancy, n (%) |  |  |  |
| Never drank | 927 (39.4) | 2354 (51.8) | <0.001 |
| Drank until pregnancy was known | 335 (14.2) | 583 (12.8) |  |
| Continued drinking occasionally | 865 (36.7) | 1342 (29.5) |  |
| Continued drinking frequently | 227 (9.6) | 267 (5.9) |  |
| Total energy intake, kcal, mean (SD) | 2058.1 (547.4) | 1997.2 (578.5) | <0.001 |
| Diet quality, score, mean (SD)^b^ | 7.7 (1.6) | 7.3 (1.7) | <0.001 |
| Family income, €/month, n (%) |  |  |  |
| <1200 | 395 (16.8) | 1355 (29.8) | <0.001 |
| 1200-2000 | 378 (16.1) | 980 (21.6) |  |
| > 2000 | 1581 (67.2) | 2211 (48.6) |  |
| Plasma TFA concentration, %, wt:wt, mean (SD)^c^ | 0.35 (0.12) | 0.36 (0.13) | <0.001 |
| **Child** |  |  |  |
| Sex (male), n (%) | 1179 (50.1) | 2308 (50.8) | 0.61 |
| HC in the second trimester, cm, mean (SD) | 17.9 (1.4) | 18.0 (1.5) | 0.14 |
| HC in the third trimester, cm, mean (SD) | 28.6 (1.2) | 28.4 (1.3) | <0.001 |
| HC growth rate, cm/week, mean (SD) | 1.09 (0.09) | 1.08 (0.1) | <0.001 |
| Data from the first imputed dataset are reported. Percentages have been rounded and may not total 100. Continuous variables were compared using analysis of variance (ANOVA) or Wilcoxon test; categorical variables were compared using chi-square test.  ^a^ Assessed using the Brief Symptom Inventory. Scores range from 0 to 4, with higher scores indicating more clinically relevant psychological symptoms.  ^b^ Assessed using a food frequency questionnaire. The overall score ranges from 0 to 15, with higher scores reflecting better adherence to Dutch dietary guidelines.  ^c^ Assessed at 20.6 (1.1) weeks of gestation.  Abbreviations: BMI, body mass index; TFA, trans fatty acid; HC, head circumference. | | | |

**Table S2. Log-transformed maternal trans fatty acids concentration during pregnancy in relation to fetal head circumference and head circumference growth**

| **Log-transformed maternal TFA concentration** | **Fetal HC at single assessments**^a^ | | | | | | **Fetal HC growth rate across assessments**^b^ **(n=6517)** | | |
| --- | --- | --- | --- | --- | --- | --- | --- | --- | --- |
|  | **Second trimester (n=6792)** | | | **Third trimester (n=6625)** | | |  |  |  |
|  | B | 95% CI | p-value | B | 95% CI | p-value | B | 95% CI | p-value |
| Model 1 | 0.05 | 0.005, 0.09 | 0.03 | -0.07 | -0.14, -0.01 | 0.03 | -0.01 | -0.02, -0.004 | 0.003 |
| Model 2 | 0.04 | -0.002, 0.09 | 0.06 | -0.10 | -0.17, -0.03 | 0.004 | -0.01 | -0.02, -0.01 | <0.001 |
| Model 3 | 0.02 | -0.02, 0.07 | 0.33 | -0.11 | -0.18, -0.04 | 0.001 | -0.01 | -0.02, -0.01 | <0.001 |
|  |  |  |  |  |  |  |  |  |  |
| **Log-transformed maternal TFA concentration** | **Total brain volume**^c^ | | | **Cortical gray matter volume**^c^ | | | **Cerebral white matter volume**^c^ | | |
|  | B | 95% CI | p-value | B | 95% CI | p-value | B | 95% CI | p-value |
| Model 1 | 19.3 | 6.7, 31.9 | 0.003 | 10.6 | 4.3, 16.8 | 0.001 | 6.5 | 0.68, 12.3 | 0.03 |
| Model 2 | 8.8 | -4.0, 21.6 | 0.18 | 5.3 | -1.1, 11.7 | 0.11 | 3.2 | -2.8, 9.2 | 0.29 |
| Model 3 | 9.0 | -3.9, 22.0 | 0.17 | 5.5 | -1.1, 12.0 | 0.10 | 3.3 | -2.7, 9.4 | 0.28 |
|  |  |  |  |  |  |  |  |  |  |
| ^a^ B’s represent difference in fetal HC (cm) per one unit increase in natural logarithm transformed maternal TFA concentration. Model 1 was adjusted for child sex and gestational age at HC assessment; Model 2 was additionally adjusted for maternal ethnicity, age at enrollment, educational level, diet quality, smoking during pregnancy, and family income; Model 3 was additionally adjusted for maternal concentrations of essential fatty acids and long-chain polyunsaturated fatty acids.  ^b^ B’s represent difference in fetal HC growth rate (cm/week) per one unit increase in natural logarithm transformed maternal TFA concentration. Model 1 was adjusted for child sex. Model 2 was additionally adjusted for maternal ethnicity, age at enrollment, educational level, diet quality, smoking during pregnancy, and family income. Model 3 was additionally adjusted for maternal concentrations of essential fatty acids and long-chain polyunsaturated fatty acids.  ^c^ B’s present volumetric difference (cm^3^) per one unit increase in natural logarithm transformed maternal TFA concentration. Model 1 was adjusted for child sex and age at neuroimaging; Model 2 was additionally adjusted for maternal age at enrollment, educational level, diet quality, smoking during pregnancy, and family income. Model 3 was additionally adjusted for maternal concentrations of essential fatty acids and long-chain polyunsaturated fatty acids. All models were weighted by inverse probability to account for attrition. The sample size for analysis was 2354.  Abbreviations: TFA, trans fatty acid; HC, head circumference. | | | | | | | | | |

**Table S3. Maternal trans fatty acids concentration (mg/L) during pregnancy in relation to fetal head circumference, head circumference growth, and child brain volume at age 9-11 years**

| **Maternal TFA concentration** | **Fetal HC at single assessments**^a^ | | | | | | **Fetal HC growth rate across assessments**^b^ **(n=6517)** | | |
| --- | --- | --- | --- | --- | --- | --- | --- | --- | --- |
|  | **Second trimester (n=6792)** | | | **Third trimester (n=6625)** | | |  |  |  |
|  | B | 95% CI | p-value | B | 95% CI | p-value | B | 95% CI | p-value |
| Model 1 | 0.01 | 0.005, 0.02 | 0.001 | -0.01 | -0.02, 0.004 | 0.24 | -0.002 | -0.003, -0.001 | 0.003 |
| Model 2 | 0.01 | 0.004, 0.02 | 0.003 | -0.01 | -0.02, -0.001 | 0.03 | -0.002 | -0.003, -0.001 | <0.001 |
| Model 3 | 0.01 | 0.001, 0.02 | 0.02 | -0.02 | -0.03, -0.01 | 0.002 | -0.002 | -0.004, -0.001 | <0.001 |
| Model 4 | 0.01 | -0.002, 0.01 | 0.14 | -0.02 | -0.03, -0.01 | <0.001 | -0.002 | -0.003, -0.001 | <0.001 |
|  |  |  |  |  |  |  |  |  |  |
| **Maternal TFA concentration** | **Total brain volume**^c^ | | | **Cortical gray matter volume**^c^ | | | **Cerebral white matter volume**^c^ | | |
|  | B | 95% CI | p-value | B | 95% CI | p-value | B | 95% CI | p-value |
| Model 1 | 2.9 | 1.0, 4.9 | 0.003 | 1.7 | 0.71, 2.7 | <0.001 | 0.9 | 0.01, 1.8 | 0.05 |
| Model 2 | 1.2 | -0.80, 3.3 | 0.23 | 0.8 | -0.20, 1.9 | 0.11 | 0.4 | -0.57, 1.3 | 0.43 |
| Model 3 | 1.3 | -0.88, 3.4 | 0.25 | 0.8 | -0.32, 1.9 | 0.16 | 0.5 | -0.50, 1.5 | 0.33 |
| Model 4 | 1.2 | -0.97, 3.4 | 0.28 | 0.8 | -0.33, 1.9 | 0.17 | 0.4 | -0.55, 1.4 | 0.38 |
|  |  |  |  |  |  |  |  |  |  |
| ^a^ B’s represent difference in fetal HC (cm) per 1 mg/L increase in maternal TFA concentration. Model 1 was adjusted for child sex and gestational age at HC assessment. Model 2 was additionally adjusted for maternal ethnicity, age at enrollment, educational level, diet quality, smoking during pregnancy, and family income. Model 3 was additionally adjusted for maternal concentrations of essential fatty acids and long-chain polyunsaturated fatty acids.  ^b^ B’s represent difference in fetal HC growth rate (cm/week) per 1 mg/L increase in maternal TFA concentration. Model 1 was adjusted for child sex. Model 2 was additionally adjusted for maternal ethnicity, age at enrollment, educational level, diet quality, smoking during pregnancy, and family income. Model 3 was additionally adjusted for maternal concentrations of essential fatty acids and long-chain polyunsaturated fatty acids.  ^c^ B’s represent volumetric difference (cm^3^) per 1 mg/L increase in maternal TFA concentration. Model 1 was adjusted for child sex and age at neuroimaging. Model 2 was additionally adjusted for maternal ethnicity, age at enrollment, educational level, diet quality, smoking during pregnancy, and family income. Model 3 was additionally adjusted for maternal concentrations of essential fatty acids and long-chain polyunsaturated fatty acids. Model 4 was additionally adjusted for maternal serum total fatty acid concentration (mg/L) to enable comparison with relative measures. All models were weighted by inverse probability to account for attrition. The sample size for analysis was 2354.  Abbreviations: TFA, trans fatty acid; HC, head circumference. | | | | | | | | | |

**Table S4. Test for non-linear relations**

| **Outcome variable** | **Sample size** | **Likelihood ratio test p-value** |
| --- | --- | --- |
| Head circumference in the 2^nd^ trimester | 6792 | 0.54 |
| Head circumference in the 3^rd^ trimester | 6625 | 0.23 |
| Head circumference growth rate from the 2^nd^ trimester to the 3^rd^ trimester | 6517 | 0.07 |
| Head circumference at birth | 3752 | 0.53 |
| Total brain volume at age 9-11 years | 2354 | 0.22 |
| Cortical gray matter volume at age 9-11 years | 2354 | 0.17 |
| Cerebral white matter volume at age 9-11 years | 2354 | 0.49 |
| Linear model was compared with non-linear model including natural splines (one knot placed at the median of trans fatty acid concentration). Model was adjusted for (gestational) age of brain assessment, sex, maternal ethnicity, age at enrollment, educational level, diet quality, smoking during pregnancy, and family income. | | |

**Figure S3.** **Natural logarithm transformed maternal trans fatty acid concentration during pregnancy per calendar time of assessment**

**
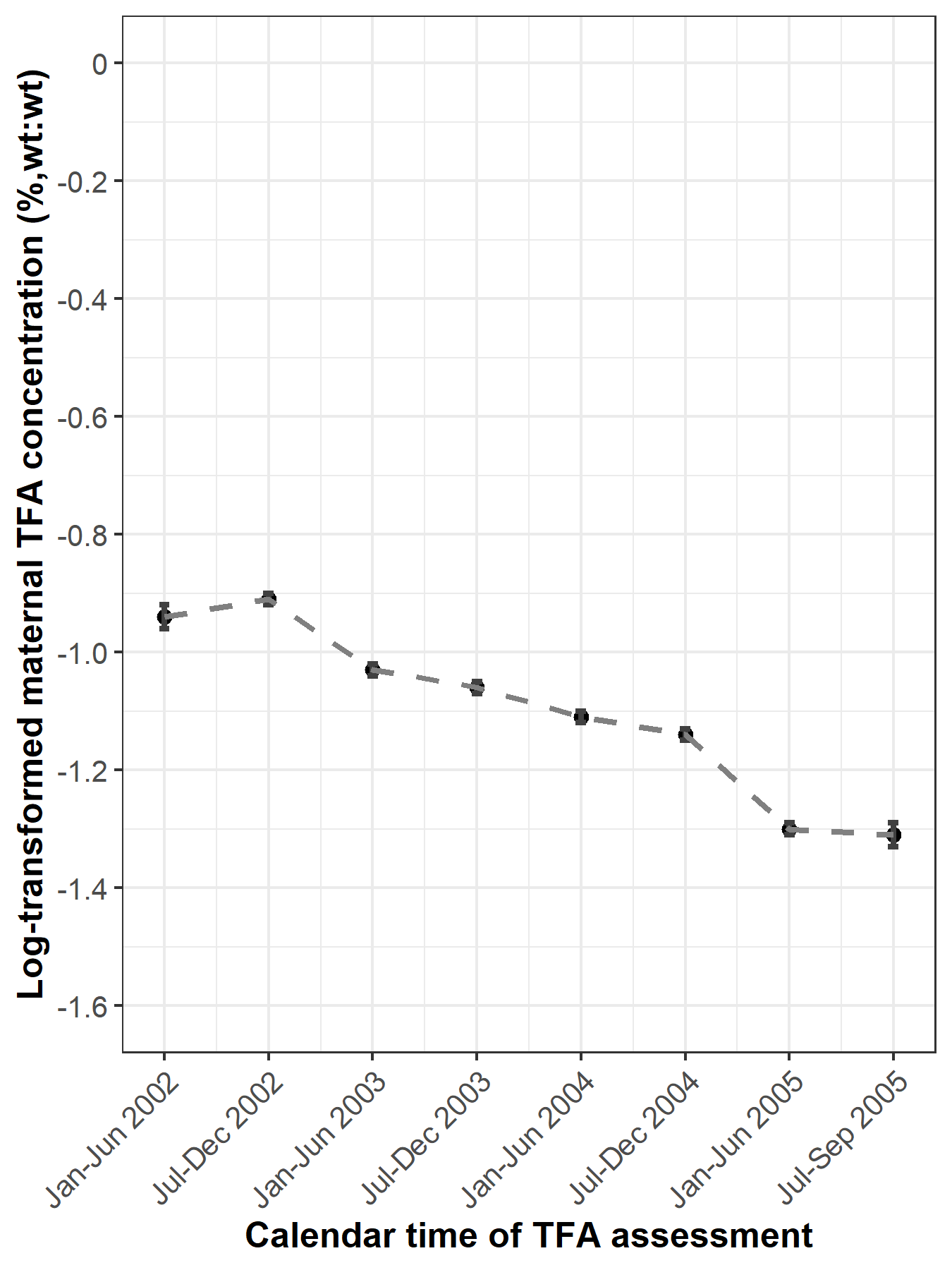
**

**Figure legend:** Natural logarithm transformed maternal TFA concentration (mean ± se) per calendar time of TFA assessment. Maternal TFA concentration was assessed in plasma at 20.6 (1.1) weeks of gestation. The Dutch initiative to further reduce TFA content in food took effect in 2003.

Abbreviation: TFA, trans fatty acid.

**Figure S4. Time trend of covariates for instrumental variable analysis (mean ± se)**

**
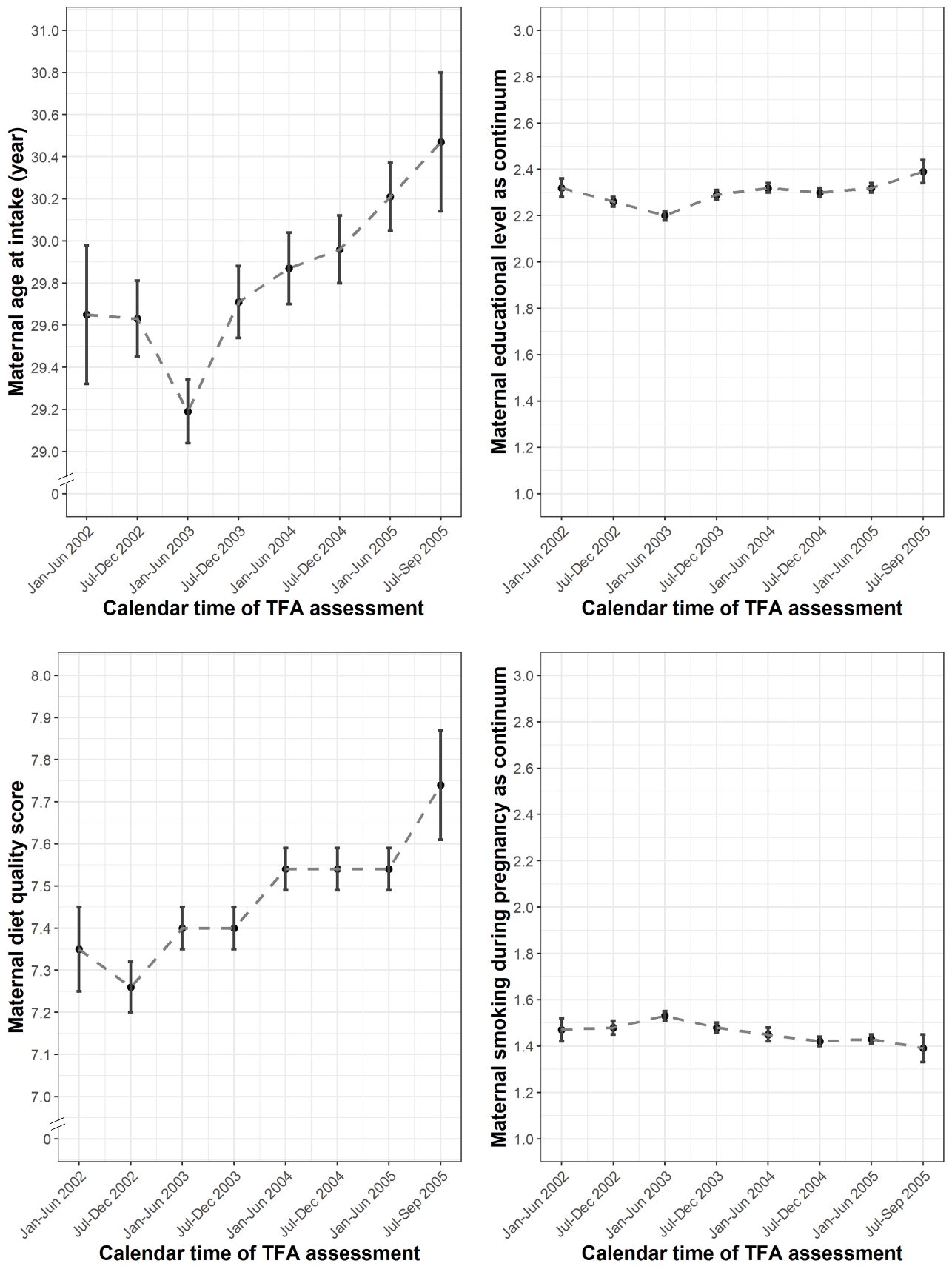
**

For illustrative purpose, categorical covariates were deemed as ordinary continuum and depicted as mean ± se. The categories were ‘1-primary or below’, ‘2-middle’, and ‘higher’ for maternal educational level; and ‘1-never smoked’, ‘2-smoked until pregnancy was known’, and ‘3-continued smoking during pregnancy’ for maternal smoking during pregnancy.

**Table S5. Maternal trans fatty acids concentration during pregnancy in relation to fetal and child head size, in participants of Dutch national origin**

| **Maternal TFA concentration** | **Fetal HC at single assessments**^a^ | | | | | | **Fetal HC growth rate across assessments**^b^ **(n=3170)** | | |
| --- | --- | --- | --- | --- | --- | --- | --- | --- | --- |
|  | **Second trimester (n=3254)** | | | **Third trimester (n=3211)** | | |  |  |  |
|  | B | 95% CI | p-value | B | 95% CI | p-value | B | 95% CI | p-value |
| Model 1 | 0.05 | -0.12, 0.22 | 0.56 | -0.53 | -0.78, -0.28 | <0.001 | -0.06 | -0.08, -0.03 | <0.001 |
| Model 2 | 0.11 | -0.06, 0.28 | 0.20 | -0.38 | -0.63, -0.12 | 0.003 | -0.05 | -0.08, -0.02 | <0.001 |
| Model 3 | 0.07 | -0.10, 0.25 | 0.41 | -0.37 | -0.63, -0.11 | 0.005 | -0.04 | -0.07, -0.02 | 0.001 |
|  |  |  |  |  |  |  |  |  |  |
| **Maternal TFA concentration** | **Total brain volume**^c^ | | | **Cortical gray matter volume**^c^ | | | **Cerebral white matter volume**^c^ | | |
|  | B | 95% CI | p-value | B | 95% CI | p-value | B | 95% CI | p-value |
| Model 1 | -25.9 | -69.0, 17.2 | 0.24 | -12.0 | -33.9, 9.8 | 0.28 | -9.2 | -29.0, 10.7 | 0.37 |
| Model 2 | -14.7 | -58.9, 29.4 | 0.51 | -6.0 | -28.3, 16.2 | 0.60 | -5.7 | -25.9, 14.6 | 0.58 |
| Model 3 | -10.4 | -55.3, 34.6 | 0.65 | -4.8 | -27.3, 17.7 | 0.67 | -3.1 | -23.7, 17.5 | 0.76 |
|  |  |  |  |  |  |  |  |  |  |
| ^a^ B’s represent difference in fetal HC (cm) per 1%, wt:wt increase in maternal TFA concentration. Model 1 was adjusted for child sex and gestational age at HC assessment; Model 2 was additionally adjusted for maternal age at enrollment, educational level, diet quality, smoking during pregnancy, and family income; Model 3 was additionally adjusted for maternal concentrations of essential fatty acids and long-chain polyunsaturated fatty acids.  ^b^ B’s represent difference in fetal HC growth rate (cm/week) per 1%, wt:wt increase in maternal TFA concentration. Model 1 was adjusted for child sex; Model 2 was additionally adjusted for maternal age at enrollment, educational level, diet quality, smoking during pregnancy, and family income; Model 3 was additionally adjusted for maternal concentrations of essential fatty acids and long-chain polyunsaturated fatty acids.  ^c^ B’s present volumetric difference (cm^3^) per 1%, wt:wt increase in maternal TFA concentration. Model 1 was adjusted for child sex and age at neuroimaging; Model 2 was additionally adjusted for maternal age at enrollment, educational level, diet quality, smoking during pregnancy, and family income. Model 3 was additionally adjusted for maternal concentrations of essential fatty acids and long-chain polyunsaturated fatty acids. All models were weighted by inverse probability to account for attrition. The sample size for analysis was 1374.  Abbreviations: TFA, trans fatty acid; HC, head circumference. | | | | | | | | | |

**Figure S5. Maternal trans fatty acid concentration during pregnancy per calendar time of assessment, in participants of Dutch national origin**


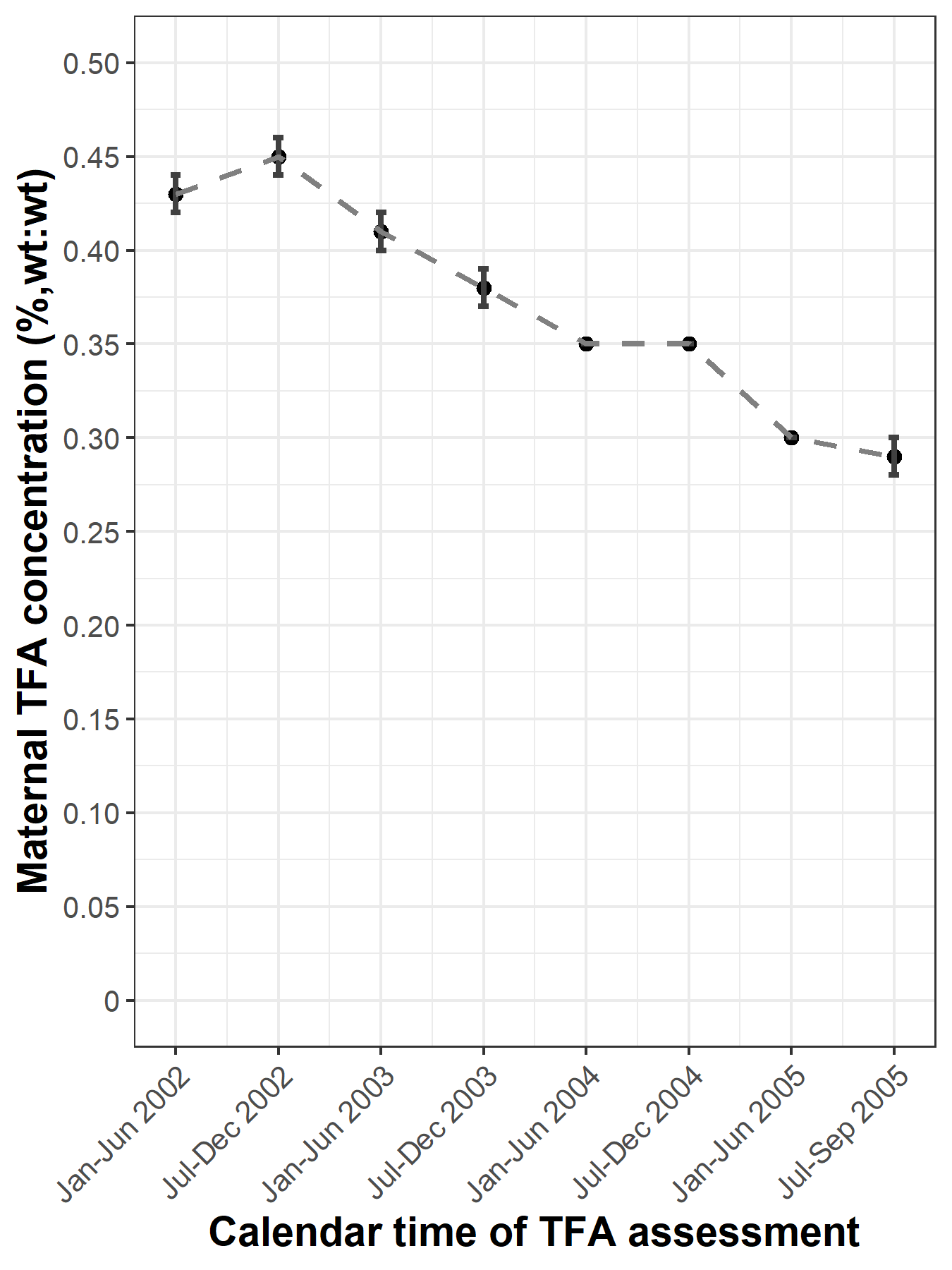


**Figure legend:** Maternal TFA concentration (%, wt:wt, mean ± se) per calendar time of TFA assessment. Maternal TFA concentration was assessed in the plasma at 20.6 (1.1) weeks of gestation. The Dutch initiative to further reduce TFA content in food took effect in 2003.

Abbreviation: TFA, trans fatty acid.

**Table S6. Instrumental variable analysis of maternal trans fatty acids concentration during pregnancy in relation to fetal HC in the third trimester and fetal HC growth rate across assessments, in participants of Dutch national origin**

| **Model** | **Fetal HC in the third trimester**^a^ | | |  | **Fetal HC growth rate across assessments**^b^ | | |
| --- | --- | --- | --- | --- | --- | --- | --- |
|  | B | 95% CI | p-value |  | B | 95% CI | p-value |
| 1 | -0.40 | -0.73, -0.08 | 0.02 |  | -0.10 | -0.12, -0.07 | <0.001 |
| 2 | -0.67 | -0.93, -0.41 | <0.001 |  | -0.09 | -0.12, -0.07 | <0.001 |
| 3 | -0.72 | -1.0, -0.45 | <0.001 |  | -0.09 | -0.12, -0.06 | <0.001 |
| Instrumental variable analysis on maternal trans 18:1 fatty acid concentration during pregnancy in relation to fetal HC in the third trimester (n=3088) and HC growth across assessments in the second and third trimesters (n=3048) in Dutch participants was performed using two-stage least squares estimation. Calendar time of maternal trans fatty acids assessment was used as the instrumental variable. The raw values of maternal trans fatty acids concentration were log-transformed to obtain a normal distribution.  ^a^ B’s represent difference in fetal HC (cm) per one unit increase in natural logarithm transformed maternal trans fatty acids concentration. Model 1 was adjusted for no covariates. Model 2 was adjusted for covariates that varied over calendar time, including gestational age at ultrasound, maternal age at enrollment, educational level, diet quality, smoking during pregnancy, and family income. Model 3 was additionally adjusted for child sex and maternal concentrations of essential fatty acids and long-chain polyunsaturated fatty acids.  ^b^ B’s represent difference in fetal HC growth rate (cm/week) per one unit increase in natural logarithm transformed maternal trans 18:1 fatty acid concentration. Model 1 was adjusted for no covariates. Model 2 was adjusted for covariates that varied over calendar time, including maternal age at enrollment, educational level, diet quality, smoking during pregnancy, and family income. Model 3 was additionally adjusted for child sex and maternal concentrations of essential fatty acids and long-chain polyunsaturated fatty acids.  Abbreviation: HC, head circumference. | | | | | | | |

**Table S7.** **Maternal trans 18:1 fatty acid concentration during pregnancy in relation to fetal head circumference, head circumference growth, and child brain volume at age 9-11 years**

| **Maternal trans 18:1 fatty acid concentration** | **Fetal HC at single assessments**^a^ | | | | | | **Fetal HC growth rate across assessments**^b^ **(n=6517)** | | |
| --- | --- | --- | --- | --- | --- | --- | --- | --- | --- |
|  | **Second trimester (n=6792)** | | | **Third trimester (n=6625)** | | |  |  |  |
|  | B | 95% CI | p-value | B | 95% CI | p-value | B | 95% CI | p-value |
| Model 1 | 0.10 | -0.04, 0.23 | 0.16 | -0.42 | -0.62, -0.22 | <0.001 | -0.04 | -0.07, -0.02 | <0.001 |
| Model 2 | 0.11 | -0.03, 0.25 | 0.12 | -0.39 | -0.59, -0.18 | <0.001 | -0.05 | -0.07, -0.02 | <0.001 |
| Model 3 | 0.05 | -0.09, 0.19 | 0.52 | -0.43 | -0.64, -0.22 | <0.001 | -0.04 | -0.07, -0.02 | <0.001 |
|  |  |  |  |  |  |  |  |  |  |
| **Maternal trans 18:1 fatty acid concentration** | **Total brain volume**^c^ | | | **Cortical gray matter volume**^c^ | | | **Cerebral white matter volume**^c^ | | |
|  | B | 95% CI | p-value | B | 95% CI | p-value | B | 95% CI | p-value |
| Model 1 | 18.1 | -21.1, 57.4 | 0.37 | 12.4 | -7.1, 31.8 | 0.21 | 6.1 | -12.1, 24.4 | 0.51 |
| Model 2 | 10.0 | -30.1, 50.0 | 0.63 | 9.1 | -11.2, 29.3 | 0.38 | 3.6 | -14.9, 22.2 | 0.70 |
| Model 3 | 9.6 | -31.3, 50.5 | 0.65 | 8.9 | -11.8, 29.6 | 0.40 | 3.7 | -15.2, 22.6 | 0.70 |
|  |  |  |  |  |  |  |  |  |  |
| ^a^ B’s represent difference in fetal HC (cm) per 1%, wt:wt increase in maternal trans 18:1 fatty acid concentration. Model 1 was adjusted for child sex and gestational age at HC assessment; Model 2 was additionally adjusted for maternal ethnicity, age at enrollment, educational level, diet quality, smoking during pregnancy, and family income; Model 3 was additionally adjusted for maternal concentrations of essential fatty acids and long-chain polyunsaturated fatty acids.  ^b^ B’s represent difference in fetal HC growth rate (cm/week) per 1%, wt:wt increase in maternal trans 18:1 fatty acid concentration. Model 1 was adjusted for child sex; Model 2 was additionally adjusted for maternal ethnicity, age at enrollment, educational level, diet quality, smoking during pregnancy, and family income; Model 3 was additionally adjusted for maternal concentrations of essential fatty acids and long-chain polyunsaturated fatty acids.  ^c^ B’s present volumetric difference (cm^3^) per 1%, wt:wt increase in maternal trans 18:1 fatty acid concentration. Model 1 was adjusted for child sex and age at neuroimaging; Model 2 was additionally adjusted for maternal ethnicity, age at enrollment, educational level, diet quality, smoking during pregnancy, and family income. Model 3 was additionally adjusted for maternal concentrations of essential fatty acids and long-chain polyunsaturated fatty acids. All models were weighted by inverse probability to account for attrition. The sample size for analysis was 2354.  Abbreviations: HC, head circumference. | | | | | | | | | |

**Figure S6.** **Maternal trans 18:1 fatty acid concentration during pregnancy per calendar time of assessment**


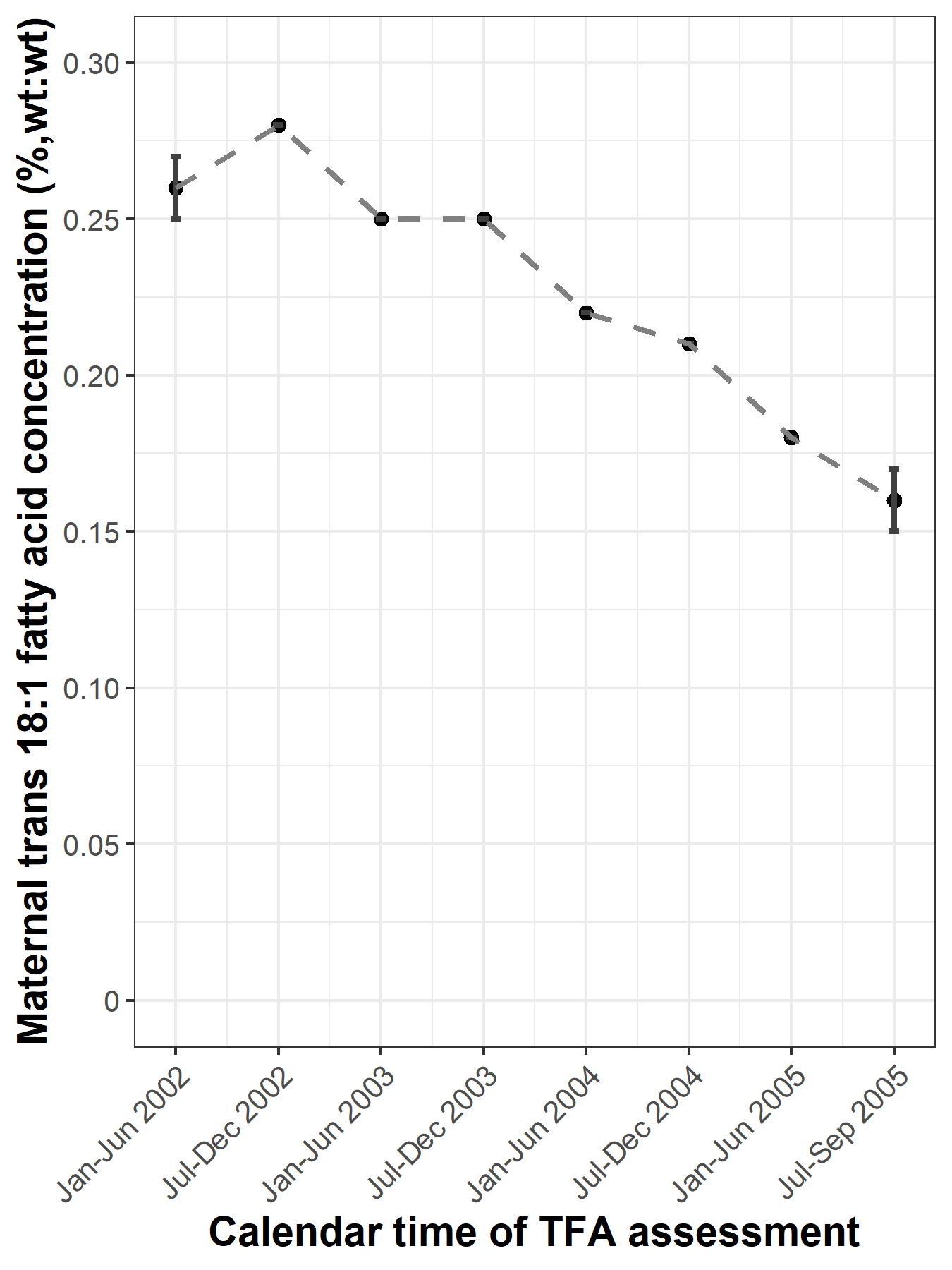


**Figure legend:** Maternal trans 18:1 fatty acid concentration (%, wt:wt, mean ± se) per calendar time of TFA assessment. Maternal trans 18:1 fatty acid concentration was assessed in plasma at 20.6 (1.1) weeks of gestation. The Dutch initiative to further reduce TFA content in food took effect in 2003.

Abbreviation: TFA, trans fatty acid.

**Table S8. Instrumental variable analysis of maternal trans 18:1 fatty acid concentration during pregnancy in relation to fetal HC in the third trimester and fetal HC growth rate across assessments**

| **Model** | **Fetal HC in the third trimester**^a^ | | |  | **Fetal HC growth rate across assessments**^b^ | | |
| --- | --- | --- | --- | --- | --- | --- | --- |
|  | B | 95% CI | p-value |  | B | 95% CI | p-value |
| 1 | -0.69 | -0.92, -0.46 | <0.001 |  | -0.10 | -0.11, -0.08 | <0.001 |
| 2 | -0.60 | -0.84, -0.37 | <0.001 |  | -0.09 | -0.11, -0.08 | <0.001 |
| 3 | -0.93 | -1.1, -0.73 | <0.001 |  | -0.11 | -0.13, -0.08 | <0.001 |
| Instrumental variable analysis on maternal trans 18:1 fatty acid concentration during pregnancy in relation to fetal HC in the third trimester (n=6383) and HC growth across assessments in the second and third trimesters (n=6280) was performed using two-stage least squares estimation. Calendar time of maternal trans fatty acids assessment was used as the instrumental variable. The raw values of maternal trans 18:1 fatty acid concentration were log-transformed to obtain a normal distribution.  ^a^ B’s represent difference in fetal HC (cm) per one unit increase in natural logarithm transformed maternal trans 18:1 fatty acid concentration. Model 1 was adjusted for no covariates. Model 2 was adjusted for covariates that varied over calendar time, including maternal age at enrollment, educational level, diet quality, and smoking during pregnancy. Model 3 was additionally adjusted for gestational age at ultrasound, child sex, maternal ethnicity, family income, and maternal concentrations of essential fatty acids and long-chain polyunsaturated fatty acids.  ^b^ B’s represent difference in fetal HC growth rate (cm/week) per one unit increase in natural logarithm transformed maternal trans 18:1 fatty acid concentration. Model 1 was adjusted for no covariates. Model 2 was adjusted for covariates that varied over calendar time, including maternal age at enrollment, educational level, diet quality, and smoking during pregnancy. Model 3 was additionally adjusted for child sex, maternal ethnicity, family income, and maternal concentrations of essential fatty acids and long-chain polyunsaturated fatty acids.  Abbreviation: HC, head circumference. | | | | | | | |

**Table S9. Maternal trans fatty acid concentration during pregnancy, fetal head circumference in the third trimester, and head growth rate in relation to child IQ at age 6 years**

| **Exposure variable** | **Child IQ** | | |
| --- | --- | --- | --- |
|  | B | 95% CI | p-value |
| **Maternal TFA concentration (n=4415)**^a^ | | | |
| Model 1 | 5.4 | 1.8, 8.9 | 0.003 |
| Model 2 | 1.1 | -2.3, 4.6 | 0.52 |
| Model 3 | 0.8 | -2.7, 4.3 | 0.66 |
|  |  |  |  |
| **Fetal HC in the third trimester (n=4299)**^b^ | | | |
| Model 1 | 1.8 | 1.3, 2.3 | <0.001 |
| Model 2 | 1.1 | 0.6, 1.5 | <0.001 |
|  |  |  |  |
| **Fetal head growth rate (n=4232)**^c^ | | | |
| Model 1 | 10.0 | 5.1, 14.8 | <0.001 |
| Model 2 | 5.7 | 1.1, 10.3 | 0.02 |
|  |  |  |  |
| ^a^ B’s represent difference in child IQ score assessed at age 6 years per 1%, wt:wt increase in maternal TFA concentration during pregnancy. Model 1 was adjusted for child sex and age at IQ assessment. Model 2 was additionally adjusted for maternal ethnicity, age at enrollment, educational level, diet quality, smoking during pregnancy, and family income. Model 3 was additionally adjusted for maternal concentrations of essential fatty acids and long-chain polyunsaturated fatty acids.  ^b^ B’s represent difference in child IQ score assessed at age 6 years per 1 cm increase in fetal HC in the third trimester. Model 1 was adjusted for child sex and age at IQ assessment. Model 2 was additionally adjusted for gestational age at HC assessment, maternal ethnicity, age at enrollment, educational level, diet quality, smoking during pregnancy, and family income.  ^c^ B’s represent difference in child IQ score assessed at age 6 years per 1 cm/week increase in fetal head growth rate between the second and third trimesters. Model 1 was adjusted for child sex and age at IQ assessment. Model 2 was additionally adjusted for maternal ethnicity, age at enrollment, educational level, diet quality, smoking during pregnancy, and family income. | | | |
